# Why do researchers decline reviewer invitations? Response to the editorial ‘The Peer Review Process: Growing Problem in Recruiting Qualified Reviewers’

**DOI:** 10.2340/aos.v84.42853

**Published:** 2025-02-03

**Authors:** Elin Hadler-Olsen

**Affiliations:** a The Public Dental Health Service Competence Center of Northern Norway, Tromsø, Norway; b Faculty of Health Sciences, UiT the Arctic University of Norway, Tromsø, Norway

In the editorial ‘The Peer Review Process: Growing Problem in Recruiting Qualified Reviewers’, Editor-in-Chief of *Acta Odontologica Scandinavica*, Palle Holmstrup, addresses the increasing difficulty in recruiting qualified reviewers for the publishing process. He cites statistics indicating that over the past decade, editors at his journal have had to double the average number of reviewer invitations to secure enough acceptances. This situation results in additional work for handling editors, prolongs the peer-review process, and causes frustration for authors awaiting the conclusion of the editorial process. Holmstrup appeals to researchers’ conscience by emphasizing the principle of reciprocity in the reviewing process.

Most researchers understand the critical role of peer review – we all need discerning eyes to identify weaknesses, errors, overinterpretations, or other issues in our manuscripts that we might have overlooked. We also recognize the frustration that arises when the process is delayed, potentially due to difficulties in recruiting reviewers. However, I am not convinced that the core issue in recruiting reviewers is a lack of awareness among researchers about the need to contribute to both sides of the reviewing process.

**Figure 1:**
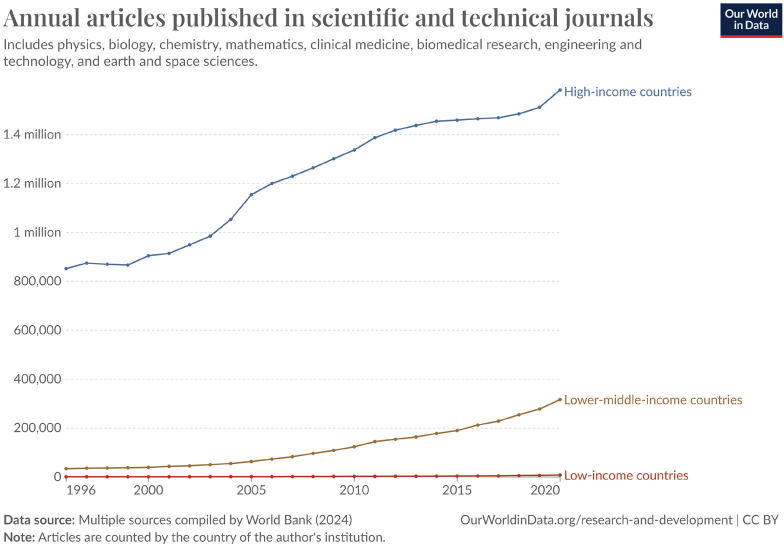
The figure is from Our World in Data: https://ourworldindata.org/research-and-development

According to Our World in Data, the number of scientific publications increased by nearly 30% between 2010 and 2020 (Figure 1). This rise may be attributed to an increase in the number of scientists, which should also expand the pool of potential reviewers. However, within the scientific community, there is a strong emphasis on publishing, often encapsulated in the phrase ‘publish or perish’. This pressure may tempt researchers to divide their work into smaller publications, known as ‘salami slicing’, and to invest less effort into each manuscript, thereby increasing the number of submissions but reducing their quality. This creates an imbalance where the volume of submissions for review grows faster than the pool of potential reviewers, straining the peer-review system.

The publishing frenzy is further fueled by the increase in predatory journals or journals with aggressive advertising strategies that drown researchers’ inboxes with requests to submit articles, serve as guest editors for numerous special issues, and conduct peer reviews. This flood of spam can wear out researchers’ willingness to contribute or overshadow requests from reputable journals.

Another potential source of reviewer fatigue is the quality of manuscripts submitted for review. My personal experience as a reviewer suggests that the overall quality of manuscripts has declined in recent years. Even though I avoid reviewing for predatory journals, a growing number of manuscripts I review appear as unpolished drafts, with confusing language, poor structure or substantial information missing. Providing thorough and constructive feedback on such manuscripts demands considerable time and effort. It also frustrates me, as I feel I am doing work that the senior authors of the manuscript should have done prior to submission. There is an increasing number of guidelines on how to conduct and report various types of research, along with free editing services and chatbots. These tools should assist researchers in planning, executing, and reporting their research adequately.

To ensure a functional peer-review system, researchers must not only be willing to serve as reviewers but also invest effort in preparing their own manuscripts well before submission. Furthermore, journal editorial offices have a responsibility to implement stricter quality checks before allowing manuscripts to proceed to review. These checks should go beyond formal requirements and include assessments of readability and adherence to quality guidelines. Artificial intelligence could enhance this process.


Best regards, Elin Hadler-Olsen


